# Evaluating the impact of prescription isodose line on plan quality using Gamma Knife inverse planning

**DOI:** 10.1002/acm2.13388

**Published:** 2021-08-17

**Authors:** Qianyi Xu, Gregory Kubicek, David Mulvihill, Warren Goldman, Gary Eastwick, Alan Turtz, Jiajin Fan, Dershan Luo

**Affiliations:** ^1^ Department of Radiation Oncology MD Anderson Cancer Center at Cooper Camden NJ USA; ^2^ Department of Radiation Oncology Inova Health System Fairfax VA USA; ^3^ Department of Neurosurgery Cooper Medical School Rowan University Camden NJ USA; ^4^ Department of Radiation Physics UT MD Anderson Cancer Center Houston TX USA

**Keywords:** Gamma Knife, isodose line

## Abstract

The impact of selection of prescription isodose line (IDL) on plan quality has not been well evaluated during inverse planning (IP). In this study, a total of 180 IP plans at five levels of IDL were generated for 30 brain metastases (BMs). For each BM, one round of IP was performed with typical IP settings, followed by a quick fine‐tuning to ensure the same target coverage and comparable conformality index. The impact of the IDL on the quality metrics (selectivity, gradient index [GI], and treatment time) was evaluated. The decrease of selectivity and increase of GI meant inferior target dose conformality and more dose spillage. Additionally, a metric directly correlated to the treatment time was proposed. For all cases, the mean GI decreased monotonically as IDL decreased from 70% to 30%, and the decreasing rate was significantly different based on tumor size. The mean selectivity and number of shots decreased monotonically as IDL decreased for all the tumors. From 70% to 30% IDL, the decreasing rate of the mean selectivity was 2.8% (*p* = 0.020), 7.7% (*p* = 0.005), and 15.4% (*p* = 0.020) and that of the number of shots was 75.4% (*p* = 0.001), 73.2% (*p* = 0.001), and 50.7% (*p* = 0.009), for the large, medium, and small tumors, respectively. For the medium and small tumor groups, the mean treatment time increased monotonically when IDLs decreased (increasing rate was 80.0% [*p* = 0.002] for medium tumors [*p* = 0.001] and 130.8% [*p* = 0.001] for small tumors from 70% to 30%). For the large tumors, the mean treatment time was the shortest at 50% IDL (59.0 min) and higher at 70% (65.9 min) and 30% (71.9 min). Overall, the GammaPlan chose smaller sectors for plans with lower IDLs except for the large size group.

## 1 INTRODUCTION

In Leksell GammaPlan (LGP version 11.1, Elekta AB, Stockholm, Sweden), the planning procedure for Gamma Knife (GK) treatment starts from defining target dose and prescription isodose line (IDL), after target volumes are drawn by physicians on magnetic resonance images. The planner can either manually place shots on the target or use the inverse planning (IP) tool to iteratively search for an optimal target dose. For the selection of IDL, 50% is a common choice by most clinics, yet great variations of IDLs have been observed. In a review^1^ by Paddick et al, the IDLs varied between 30% and 94% in treating vestibular schwannoma, when multiple shots were used during forward planning (FP).

Paddick et al found that the IDL of a GK plan was correlated to the amount of low dose spillage in the plan.^1^ For the same treatment target, the authors performed three forward plans with the same conformality index (CI). The CI was a measure of how well the target dose was conformed to the target shape.^2^ Despite the same target dose conformality, the low dose spillage, considered as one of the major causes of radiation necrosis, was significantly different in three plans. When comparing these plans, a question was raised immediately of which IDL was optimal and corresponding to the lowest low dose spillage. For 50 treated plans with multiple shots, the authors re‐prescribed the target dose at different IDLs (ranging from 20% to about 70%) and examined the optimal IDL in terms of low dose falloff. The gradient index (GI), defined as the ratio of isodose volume of the half prescription dose to that of the prescription dose, was used to evaluate low dose falloff at the half of the prescription dose.^1^ However, simply re‐prescribing a plan could significantly compromise the conformality of the target dose, and the quality of the re‐prescribed plans would not be acceptable for clinical treatment. It would be more desirable to compare GK plans at different IDLs with acceptable plan quality. This was very difficult to achieve in Paddick's study, because manual planning was the only choice in GammaPlan with versions earlier than 10; hence, it was not feasible to generate numerous FP plans with consistent planning quality. IP has been developed and reported by multiple groups^3^, ^4^, ^5^, ^6^, ^7^, ^8^, ^9^ but not available until LGP version 10.0 was released in 2010. Under the IP feature, the target coverage, CI, GI, and treatment time could be optimized concurrently based on predefined planning settings. As evaluated by Schlesinger et al, the IP tool in the GammaPlan could generate plans with more consistent quality and in consequence,^10^ so it is feasible to quickly generate multiple plans at different IDLs with acceptable quality. This enabled feasibility of evaluating the impact of the IDL on the quality of GK plans.

We generated IP plans at five levels of IDL for 30 brain metastases (BMs) of various sizes, each with the same target coverage and similar CI. The impact of the IDL on the quality of the plan, for example, GI, selectivity, number of shots, and treatment time, was evaluated. The decrease of selectivity and increase of GI mean worse target dose conformality and worse low dose spillage at half of the prescription dose, respectively. We further examined the size of the sectors selected by the IP optimizer in relation to the corresponding plan quality. Finally, we evaluated the impact of the numbers of starting shots for IP on plan quality for certain IDLs. As IP has started to gain popularity for GK planning, we expected that the findings from our study would be useful for GK planning when selecting IDL for tumors with various sizes.

## 2 METHODS AND MATERIALS

### 2.1 Patient selection

Under the approval of the Institutional Review Board, 30 patients with a total of 30 BMs treated on the LGK Perfexion system were retrospectively recruited in the study. The selection criterion was based on the tumor size (the maximum size in one dimension) with 30 patients being equally divided into three groups: large tumors (>2 cm), medium tumors (between 1 and 2 cm), and small tumors (between 0.5 and 1 cm). Tiny tumors (<0.5 cm) were not included in the study, as a single shot was sufficient to cover the target and no IP was needed for planning. All BMs were treated in a single fraction. The delivered dose (in mean ± standard deviation [SD]) was 17.5 ± 1.9 Gy for the 10 large tumors and 20 Gy for the medium and smaller BMs. The sizes of the large, medium, and small tumor groups were 2.56 ± 0.22, 1.50 ± 0.20, and 0.86 ± 0.15 cm, and corresponding volumes were 5.36 ± 0.12, 1.26 ± 0.54, and 0.38 ± 0.19 cm^3^, respectively. None of the tumors had immediately adjacent organs at risk (OARs). About 70% of the patients treated in our center had no abutting OARs based on a review of patient data in the past 2 years.

### 2.2 IP at different IDLs

The functionality of the IP tool was well described by Schlesinger et al.^10^ For each patient, IP plans were generated at individual IDL of 30%, 40%, 50%, 60%, and 70%, which covered the common range of IDLs for GK planning. The planning process started from defining the target dose and the corresponding IDL at the Target tab in GammaPlan. Shots were filled automatically based on the Fill setting, and composite shots were allowed. The same Fill setting was used for the same tumor at different IDLs. In the Optimization tab, the IP parameters, including coverage, selectivity, GI, and beam‐on, were set to 0.81, 0.19, 0.1, and 0.19 for the large and medium tumor groups. For small tumor group, only the coverage parameter was increased slightly to 0.83. The definition of these parameters could be found in the white paper.^11^ For each plan, one round of optimization was performed, which typically took 2–3 min. The IP objective was defined as a combination of weighted coverage, selectivity, GI, and treatment time.^11^ The iteration was stopped by the user when either the objective stabilized or the number of iterations reached 8000. After optimization, missing target coverage was often observed in the regions with abrupt anatomy changes, for example, the most superior and inferior slices of the target. One to three shots were manually added to ensure adequate target coverage in the regions. For a fair plan comparison, we ensured the same target coverage for the same tumor at different IDLs. For each group of tumors, the selectivity, GI, treatment time, and number of shots were evaluated at different IDLs.

Similar to the segments in conventional intensity‐modulated radiation therapy (IMRT) plans, we noticed that the size of the sectors selected by the optimizer during optimization was a major indicator of the plan quality, treatment time in particular. For individual shot in an IP plan, the collimator sizes of the eight sectors were often different due to the non‐sphere shape of the tumor. The mean sector size of an IP plan was highly correlated to the quality of the plan when evaluating the IDL impact. As beam‐on time for each shot reflected its weight in an IP plan, we proposed the time‐weighted mean sector size for plan quality evaluation:

$$\bar{S}=\sum_{i=0}^{N}\text{mean}\left(S_{i}\right)*T_{i}/T_{\text{total}},\left(1\right)$$

where mean (*S_i_*) was the mean size of the sectors for shot *i*, *T_i_* was beam‐on time for shot *i*, *T*_total_ was the total beam‐on time for the plan, and *N* was the total number of shots. The mean(*S_i_*) is calculated from the shot pattern of shot *i*. For example, for a shot pattern of [4 16 8 8 B 16 B 8], the mean size will be 7.5 (=(16 × 2 + 8 × 3 + 4 × 1)/8). The number in the brackets was the size (unit of millimeter) of the individual collimator in the corresponding sector, and a shot was composed of eight sectors. The letter B meant the individual collimator was blocked. All the parameters employed to calculate $\bar{S}$ were from the plan report, and $\bar{S}$ was calculated for all the plans.

We also evaluated the impact of the starting shots on the quality of IP plans. The initial shots could be either manually filled by the planner or automatically filled by the GammaPlan. For automatic filling, the number of the initial shots was determined by the setting of collimator size under IP option. For a fair planning comparison, we chose automatic filling with the same filling setting for all the plans. It was noticed that the number of filled initial shots was correlated to the IDL: Lower IDL led to fewer initial shots. For example, the initial shots for plans at 30% IDL were significantly fewer than others. To evaluate the effect of the initial shots on plans at 30% IDL, we performed another round of independent planning on those plans by forcing the same starting shots as in those planned at 50% IDL. This was achieved by choosing 50% IDL when starting planning and filling in the same number of initial shots for 50% IDL plans. Before optimization was started, IDL was switched back to 30% under prescription. The resulting plans were compared with the plans at 30% IDL with fewer starting shots.

We performed statistical analysis for our results (IBM, SPSS Statistics, version 24). To compare means, paired sample *t* tests were performed for dependent data groups. A *p* value <0.05 was considered statistically significant.

## 3 RESULTS

**FIGURE 1:**
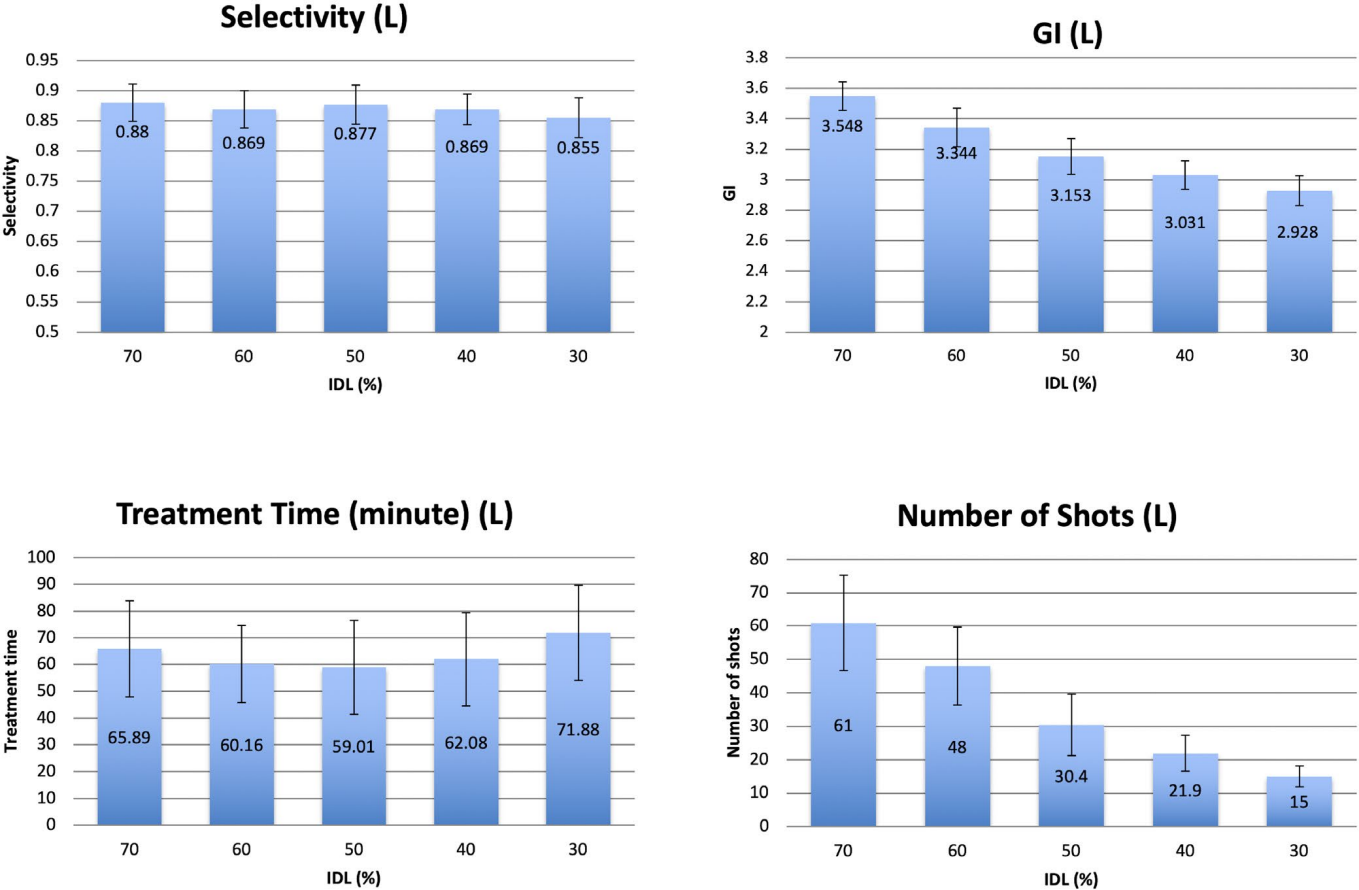
The means of selectivity, gradient index (GI), treatment time, and number of shots for large tumor group. Abbreviation: IDL, isodose line

**FIGURE 2:**
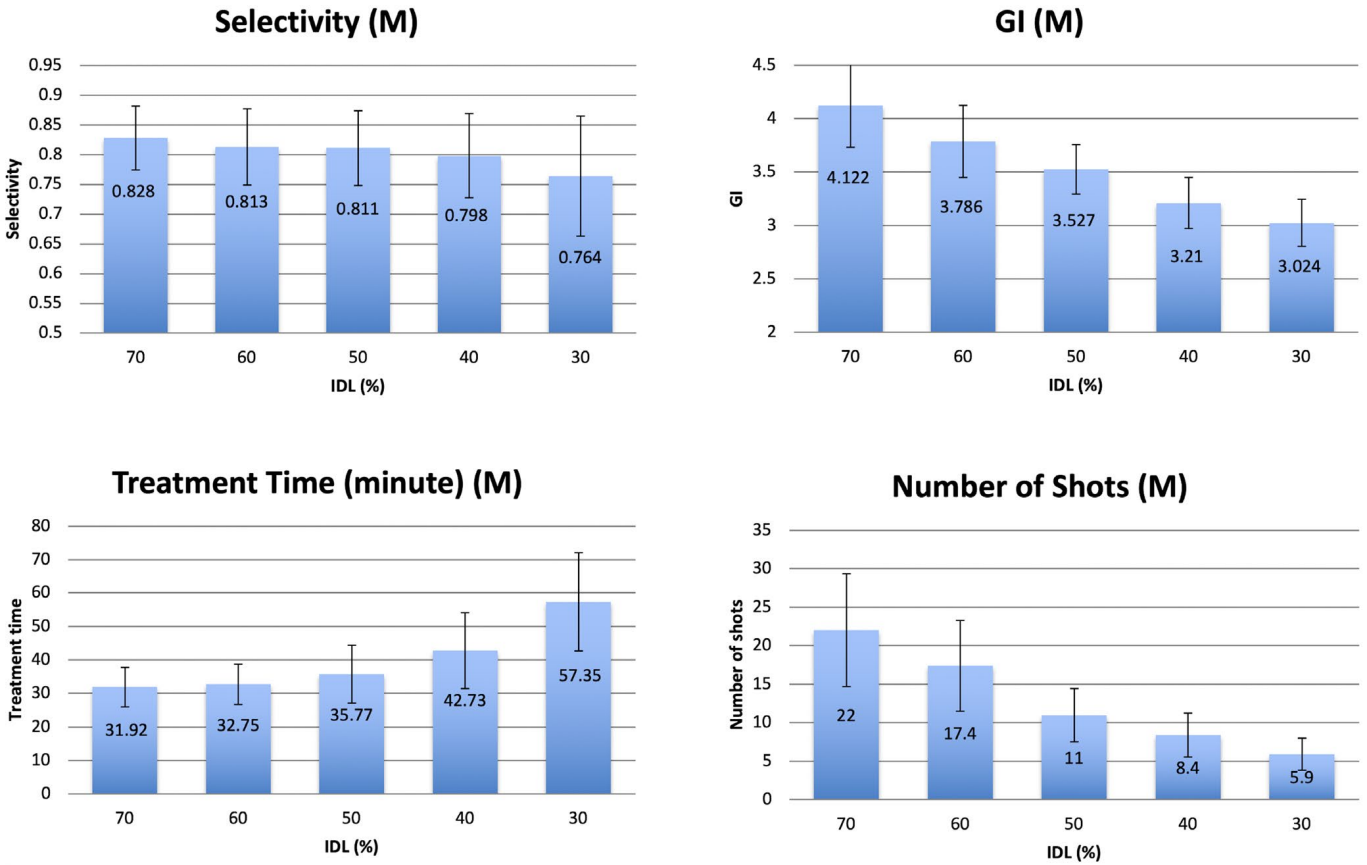
The means of selectivity, gradient index (GI), treatment time, and number of shots for medium tumor group. Abbreviation: IDL, isodose line

**FIGURE 3:**
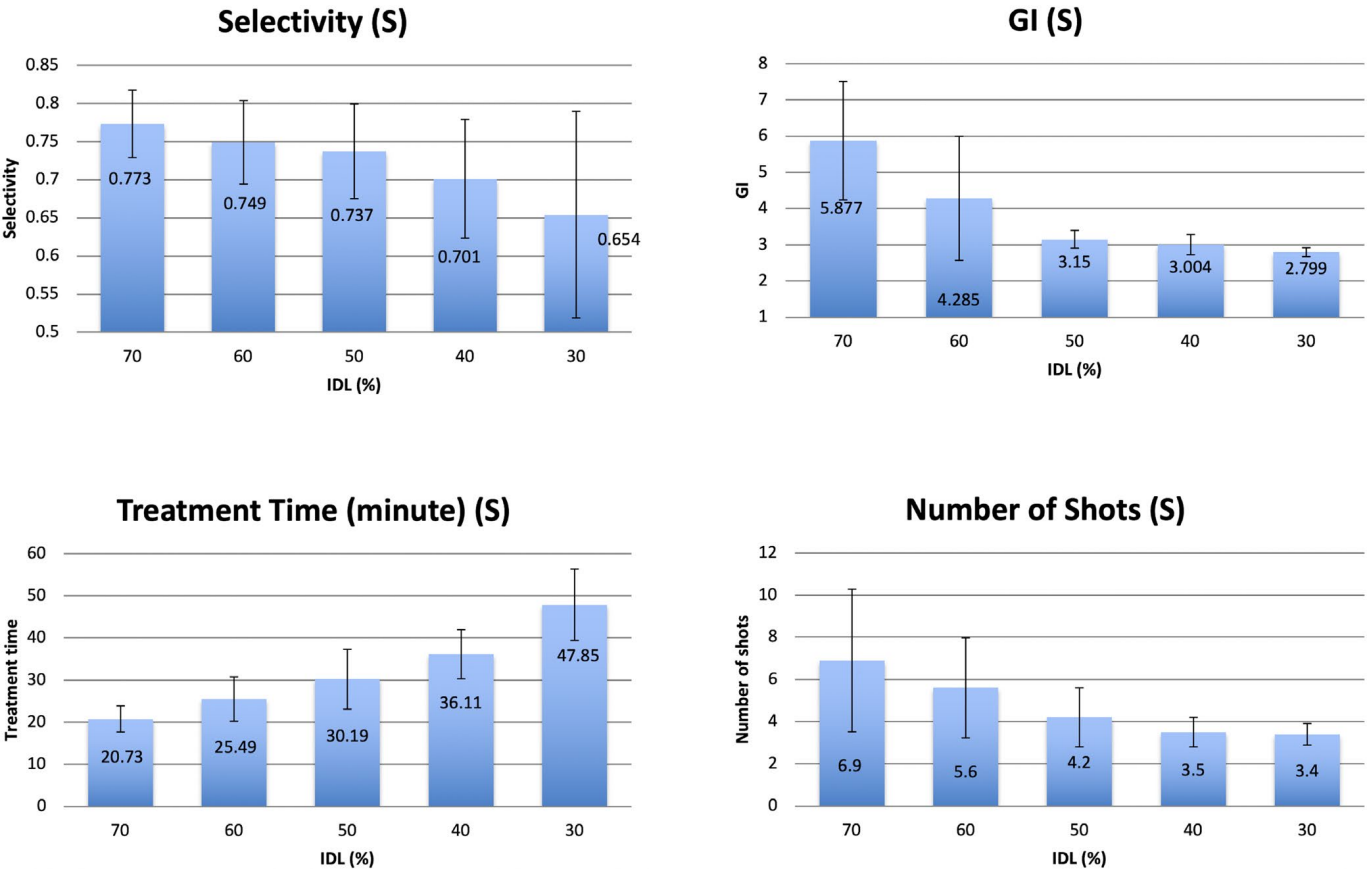
The means of selectivity, gradient index (GI), treatment time, and number of shots for small tumor group. Abbreviation: IDL, isodose line

Not counting the 30 additional plans for the 30% IDL with identical starting shots to the 50% IDL, a total of 150 plans were generated through IP for 30 brain tumors at five levels of IDL. The overall mean and SD of the coverage, selectivity, GI, and treatment time for all the plans were 98.0 ± 0.8%, 0.80 ± 0.09, 3.5 ± 0.9, and 45.3 ± 19.5 min, respectively. For each patient, the coverage was the same for all the plans at different IDLs. The means of selectivity, GI, treatment time, and number of shots for three tumor groups were compared at different levels of IDL (Figures 1, 2, 3). The error bars meant ±1 SD. For all cases, the mean GI decreased monotonically as IDL decreased from 70% to 30%, and low dose spillage was improved correspondingly. However, the decreasing rate was significantly different in terms of the size of the tumors. Using the mean GI at 70% IDL as the baseline, the GI at 30% IDL was decreased by 17.6% (*p* = 0.001), 26.6% (*p* = 0.001), and 52.4% (*p* = 0.001) for the large, median, and small tumor groups, respectively. The mean selectivity decreased monotonically as IDL decreased for all the tumors; that is, the conformality of the target dose became worse. The decreasing rate from 70% to 30% IDL plans was 2.8% (*p* = 0.020), 7.7% (*p* = 0.005), and 15.4% (*p* = 0.020) for the large, median, and small tumors, respectively. The number of shots decreased monotonically from high‐IDL to low‐IDL plans for all the tumors. The mean decreasing rate from 70% to 30% IDL plans was 75.4% (*p* = 0.001), 73.2% (*p* = 0.001), and 50.7% (*p* = 0.009) for the large, median, and small tumors, respectively. For the medium and small tumor groups, the mean treatment time increased monotonically when IDLs decreased from 70% to 30%, and the increasing rate was 80.0% (*p* = 0.002) and 130.8% (*p* = 0.001), respectively. For the large tumors, the mean treatment time was the shortest at 50% IDL (59.0 min) and higher at 70% (65.9 min) and 30% (71.9 min). The treatment time at 70% and 30% IDL plans was 11.7% (*p* = 0.009) and 21.9% (*p* = 0.001) higher than that at 50% IDL plans.

**FIGURE 4:**
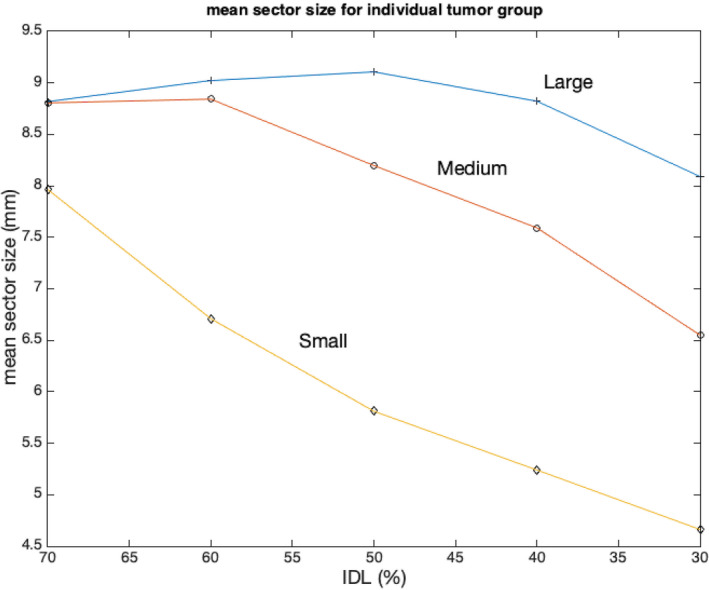
The mean sector size for all three tumor groups at different isodose lines (IDLs)

The time‐weighted mean sector size for three groups of tumors at different IDLs was plotted in Figure 4. Overall, the GammaPlan chose smaller sectors for plans with lower IDLs. For the small and medium size groups, the mean sector size decreased monotonically when IDL decreased. For the large size group, the mean sector size was the largest at 50% IDL and smallest at 70% IDL. At 70% IDL, the mean sector size was not significantly different between three groups (8.8 mm for large and medium groups and 8.0 mm for small group). But at 30% IDL, the mean sector sizes were very different between groups (8.1 mm for large tumor group, 6.5 mm for medium tumor group, and 4.7 mm for small tumor group).

The increased number of starting shots did not improve the quality of plans at 30% IDL significantly (Table 1). For seven tumors in the small group, the number of initial shots was the same between 30% and 50% IDL plans, so they were not included in this section. The rest of the three small tumors were added to the medium group. The number of initial shots was almost doubled in the replanned cases. For the large tumor group, the mean selectivity was improved by 0.01 (*p* = 0.129), the mean GI was worsened by 0.06 (*p* = 0.124), and treatment time increased by 5.8% (*p* = 0.349) after increasing the number of the initial shots. For the medium group, the mean selectivity was improved by 0.04 (*p* = 0.079), the mean GI was worsened by 0.04 (*p* = 0.521), and treatment time increased by 5.1% (*p* = 0.260). For all patients, the mean selectivity was improved by 0.03, the mean GI was the same, and mean treatment time increased by 5.5% after the number of the initial shots was increased. Overall, the number of the initial shots had minimal impact on the final quality of the plans at 30% IDL.

**TABLE 1:** Comparison between the original plans and plans with increased number of initial shots

Original plans				Plans with increased initial shot number			
Selectivity	GI	Shots	Time	Selectivity	GI	Shots	Time
0.86 ± 0.03	2.93 ± 0.10	15 ± 3.09	71.88 ± 17.83	0.87 ± 0.04	2.99 ± 0.13	30.4 ± 9.19	76.06 ± 18.10
0.74 ± 0.11	2.98 ± 0.22	5.31 ± 2.14	54.23 ± 14.29	0.78 ± 0.06	2.94 ± 0.18	9.69 ± 3.64	56.99 ± 11.10
0.79 ± 0.10	2.96 ± 0.18	9.52 ± 5.53	61.90 ± 17.93	0.82 ± 0.06	2.96 ± 0.16	18.69 ± 12.32	65.28 ± 17.17

Note

The unit of time was minute. The top row was the large tumor group, the middle row was the medium tumor group, and the bottom row was all the tumor groups.

Abbreviation: GI, gradient index.

## 4 DISCUSSION

We performed an extensive planning study to evaluate the impact of the IDL on the quality of GK IP plans based on a commercially available treatment planning system. The impact of the IDL on the plan quality has been discussed in other cone‐based systems, for example, CyberKnife,^12^, ^13^ but rarely for GK. Johnson et al evaluated the IDL selection on the quality of GK plans, and the number of shots was limited up to two based on their shot within shot technique.^14^ In both CyberKnife and GK systems, the flattening filter‐free beam had a spike peaked in the middle of the beam profile. The rate of the lateral dose falloff was mostly determined by the gradient of the beam profile, and the rate of the longitudinal dose falloff mainly followed the gradient of the beam percentage depth dose curve. Overall, the lateral dose falloff was significantly sharper than that in the longitudinal direction and therefore a more determinant factor for the quality of the plans. The selection of IDL requested which part of the beam profile was preferred to paint the target dose. The shape of GK profile has been similar to those of flattening filter‐free beams in the cone‐based system, and the same rational should follow.

**FIGURE 5:**
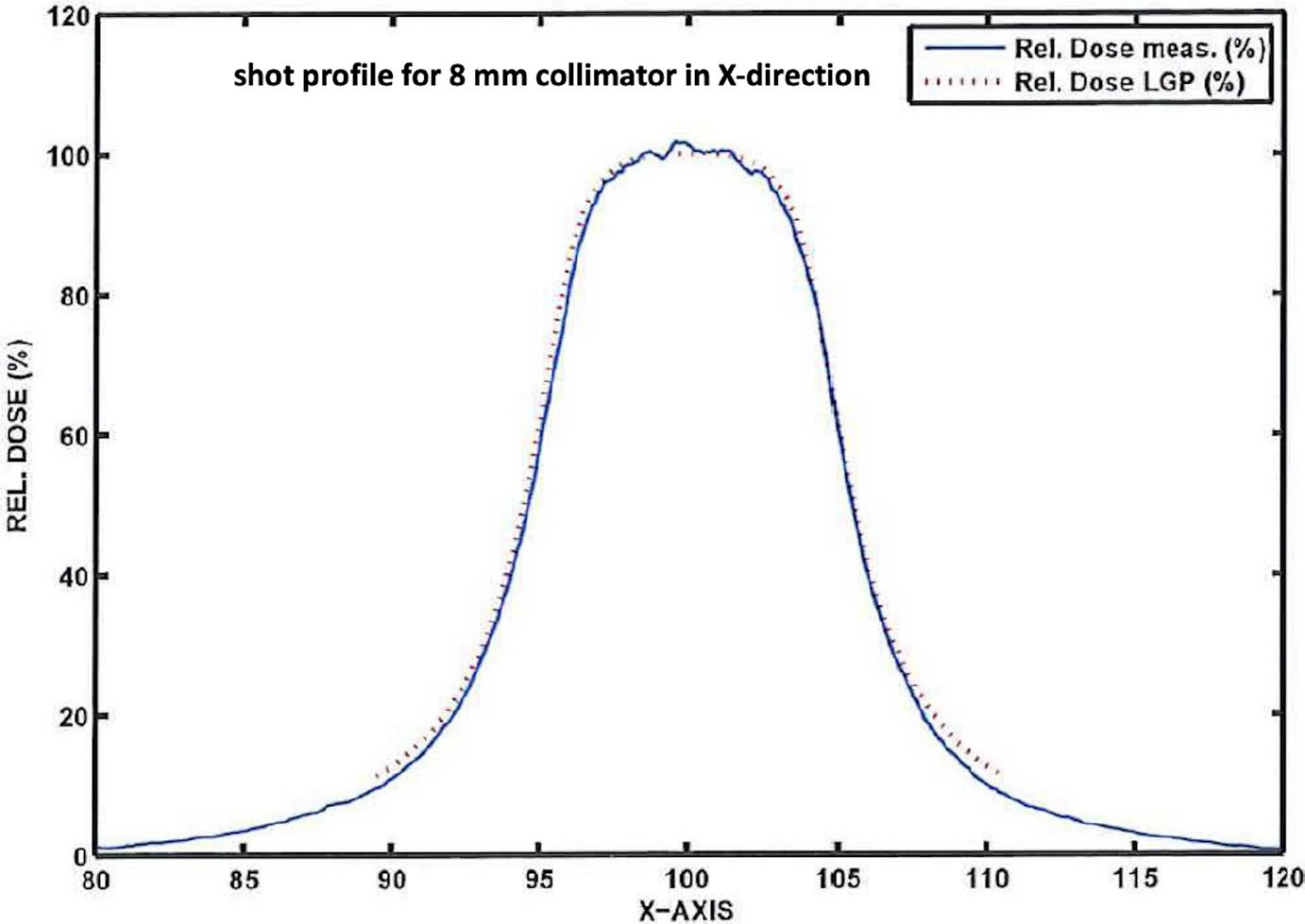
Gamma Knife shot profile in X‐direction for 8‐mm collimator. The figure was copied from Elekta ICON acceptance report, and the solid profile was measured using film by Elekta. Abbreviation: LGP, Leksell GammaPlan

An example of a GK shot profile was shown in Figure 5 (8 mm in X‐direction with full width at half maximum [FWHM] of 10.82 mm). We started from a conceptual 1D single shot to explain the impact of IDL selection for GK planning. The 1D single shot assumed a simplified geometry that beam entered from the anterior direction, and dose in a coronal plane of the target was evaluated, without considering any scatter and leakage. Also, 1D meant the evaluated dose in any axis of the coronal plane because the shot was circular symmetry. If lower leakage dose to the left side of the target (assuming shot and target were overlapping) was desired, a lower IDL was preferred because that leakage dose would be obviously minimal. However, the width of the shot profile inside the target also had to be considered as the shots were always symmetric. If a very low IDL was chosen to minimize dose spillage, the width of the shot profile at that IDL would be increased significantly, which might increase dose spillage to the right side of the target. As the size of tumor in GK stereotactic radiosurgery was often small, simply requesting low IDL and pushing for better GI might not always be feasible. Meanwhile, forcing the optimizer to choose a smaller collimator might help. However, even the smallest shot (4 mm) had the size limitation. The FWHM for the 4‐mm collimator in the X‐direction was 6.07 mm, and the shot profile width was about 8.25 mm at 30% IDL. Due to this physical limitation, it would be more difficult to use lower IDL for smaller targets while maintaining a reasonable CI. Note that the example discussed here was for a 1D single collimator case. For clinical cases, the overall dosimetry effect would be more comprehensive, and more factors should be included. For example, the target might not be in the middle of the brain, and shots from all directions might have different attenuations. In addition, the shot profile is slightly different in Z‐direction than in the other directions. (The FWHM in Z is smaller than that in X‐ or Y‐direction.) As different collimator size may be employed in different sectors in each shot, the final target dose would have a sector overlaying effect due to combination of different beam sectors. But the same rational should follow that lower IDL led to lower dose spillage outside of the target, which was consistent with what we have found in this dosimetry study.

We also noticed that if higher selectivity was desired, higher IDL would be preferred because the width of the portion of the shots to paint the dose would be narrower (beam throughput was also reduced). The concept was similar to conventional IMRT planning in that higher number of smaller beam segments would be preferred for a more conformal target dose. However, on the basis of the shot profile in Figure 5, the dose spillage outside the target in this case would increase significantly. This explained why the selectivity and GI were competing with each other at all IDLs for all the cases. It was mentioned that the dropping rate of selectivity between IDLs varied depending on tumor size, and the dropping rate was the lowest in the large tumor group. The large tumor group had the size advantage over other groups in that a larger number of shots of different collimator sizes allow for more combinations utilized at different IDLs. In other words, larger shots could provide great throughput, and smaller shot could paint more conformal dose. At 30% IDL, the mean number of shots was 15.0 ± 3.1 for the large group, which was significantly higher than those of the medium (5.9 ± 2.1) and small (3.4 ± 0.5) tumor groups. With the best GI at 30% IDL, the selectivity for the large tumor group was still reasonable (only 2.9% lower than the best selectivity at 70% IDL). At 50% IDL, the IP plan for the large tumor group was the most balanced in terms of selectivity and GI (the mean selectivity was 0.3% lower than the best mean selectivity, and the mean GI was 7.1% higher than the best mean GI). One other factor that could potentially affect the quality of the plan was the gradient of the profile. From Figure 5, we could observe that the gradient of the profile was highly depending on the selected IDL. Johnson et al reported for a single shot setting; the dose gradient in axial plane was the steepest at 56%–63%, 62%–70%, and 77%–84% IDL for 4‐, 8‐, and 16‐mm cones, respectively, based on their shot within shot technique.^15^ Unlike other planning systems, shells currently are not allowed in this version of GammaPlan, and only GI (the dose falloff at 50% of the prescription) was incorporated in the optimization, which was distal to the target location. In the future, the effect of the gradient of profile on the plan quality should be investigated when immediate dose falloff was included as the part of optimization, for example, shell constraints.

Another important factor for GK IP planning was the treatment time. The impact of the treatment time on the GK treatment has been no different from linac treatments. Lengthy treatment led to patient discomfort, inconvenience, and more treatment uncertainties due to motion, especially for frameless ICON treatment. We found that the treatment time increased significantly when IDL became lower for medium and small tumor groups. As shown in Figure 5, the shot size (width of shot profile) became wider at lower IDL, and much smaller collimators had to be used to maintain CI in the medium and small tumor groups for this IDL. This explained why the time‐weighted mean sector size was much smaller for the small and medium tumor groups at 30% IDL. In consequence, the treatment time became much longer due to lower throughput from the smaller collimators. We had to point out that the number of shots was inversely correlated to the treatment time between IDLs for these two groups of tumors; for example, the number of shots was the highest at 70% IDL, and treatment time was actually shortest. As stated in the prior paragraph, more shots were needed in higher IDL plans to achieve reasonable selectivity, also due to the lower beam throughput at high IDL. For these two groups of tumors, the mean shot numbers at 70% and 30% IDLs were 6.9 versus 3.4 (small size) and 22 versus 5.9 (medium size), respectively. For GK delivery, shot transition time, including couch movement and source movement between shot and block locations, was overall small, comparing with beam‐on time of each shot. Thus, the effect of smaller time‐weighted sector played a more vital role in determining treatment time and longer treatment time for 30% IDL plans warranted, comparing with 70% IDL plans. Unlike for the small and median tumor groups, requesting for shots of smaller size at lower IDL was less critical in IP for the large tumor group. As a result, more constant mean sector size was observed between different IDLs in Figure 5. However, the treatment time was still directly correlated to the mean sector size, as confirmed in the small and median tumor groups. The time‐weighted mean sector size at 50% IDL was the largest (9.1 vs. 8.8 mm at 70% IDL and 8.1 at 30% IDL). Accordingly, the mean delivery time at 50% IDL was the shortest (11.7% and 21.8% shorter than time at 70% and 30% IDLs, respectively).

## 5 CONCLUSIONS

We extensively evaluated the impact of the prescription IDL on the quality of GK IP plans in terms of GI, selectivity, number of total and starting shots, sector size, and treatment time and expect that it will help GK users to decide IDL for GK IP. For the large tumor group, 50% IDL was recommended as it achieved the most balanced plan in terms of GI, selectivity, and treatment time. For the medium and small tumor groups, the GK users should decide preferred plan parameters in order of priority and chose the IDL based on findings in our study.

## CONFLICT OF INTEREST

N/A

## AUTHOR CONTRIBUTIONS

Q. X. and D. L. contributed to the conception, design, and analysis of the study. The first draft of the manuscript was written by Q. X., and the rest of the authors commented and edited the manuscript. We confirm that all coauthors contributed to the study.
